# Death of Theodore D. Buhl

**Published:** 1907-05

**Authors:** 


### DEATH OF THEODORE D. BUHL.

Theodore D. Buhl, president of the Parke, Davis & Company, died suddenly April the 7th, and we desire to extend our deepest sympathy to his relatives, friends and associates. We publish the following memoriam which was adopted by the Board of Directors on behalf of the stockholders and employes and well expresses the high esteem in which he was held by all who knew him.

"Ten and a half years ago Theodore D. Buhl cast in his lot with this house. Throughout that period he has given us the benefit of his large experience, his sound judgment, his great power in the commercial world, his granite credit reared on an unwavering honesty. As president of the house he was the perfect type of integrity and fidelity to all the stockholders. His high sense of duty as a trustee pledged to administer the property and guard the interest of others, was ever uppermost in his thoughts.. The peculiar responsibilities and hazards of our work—our obligations as purveyors to the medical profession and to suffering humanity, were to him always a solemn appeal. The ultimate triumph of character in business was with him a conviction as deep and strong as instinct. The remote future and the distant prize concerned him more than the present gain.

"The strength which he gave this house and all the many enterprises in which he shared, signally exhibits what the world should realize especially at this hour—that rich men of unflinching honesty and sound judgment are of inestimable value to their communities. They are the employers of labor, the authors of new industries, the creators of new values, the pioneers who open up vast avenues of opportunity for their followers. As they succeed or fail, the comfort, the very bread, of thousands is assured or endangered. We hear much these days of unscrupulous, predaceous wealth, but what of the type of Theodore Buhl—what of the men who consider the trust of their fellowmen the best of their possessions, who have a horror of stock-jobbing methods, who never seek an unfair advantage, who never lend their names to a dubious enterprise?

"As a director, Mr. Buhl was the soul of courtesy, kindness and deference. As an employer he was considerate, thoughtful, mindful of the comfort, interests and claims of his employes. To their grievances he gave always a patient and attentive ear. He encouraged the manly expression of honest opinion, and when it differed from his own his effort was to convince and persuade, not to invoke his authority or impose his will.

"On behalf of the stockholders, employes and executives of Parke, Davis & Company, we record this testimony to the lasting service rendered us by our lamented president. To the members of the bereaved family we offer our warm and heartfelt sympathy. May strength be theirs to bear their sorrow. May they find much comfort in the memory of a life rich in well-doing and in good repute."

---

Ernest von Bergmann, an eminent surgeon and leader of the medical profession in Germany, died at Weisbaden, March 25, after an operation for appendicitis, aged 70.

There are few surgeons more widely known throughout the world to1day than was von Bergmann. He was a voluminous and successful writer, a deep thinker, an able, skilful surgeon and tireless in his efforts to promote the welfare of the medical profession.

---

A recent and very plausible theory ascribes rheumatism:—"to toxines formed in the alimentary canal as the result of disordered digestive functions, producing disturbances in metabolism and alteration in the tissues. The body suffering these effects of autointoxication has its vital resistance lowered and is therefore subject to microbic invasion."

Tongaline from the character of its composition has an antitoxic effect on these microbes and by its stimulating action on the liver, the bowels, the kidneys and the pores, it eliminates promptly and thoroughly the poisonous germs which are the cause of rheumatism, neuralgia, grippe, gout, nervous headache sciatica, lumbago, tonsilitis and heavy colds.

